# The Maybrick Case Again

**Published:** 1903-04

**Authors:** 


### *EDITORIAL NOTES AND COMMENTS.*

The Business office of The Journal-Record is No. 64 Marietta Street.
The Editorial office is 318-319-320 The Prudential.
Address all Business communications to Dr. M. B. Hutchins, Mgr.
Make remittances payable to The Atlanta Journal-Record of Medicine.
On matters pertaining to the Editorial and Original Communications address Dr. Bernard Wolff, Atlanta.
Reprints of original articles will be furnished at cost price. Requests for the same should always be made *on the manuscript*.
We will present, post-paid, on request, to each contributor of an original article, twenty (20) marked copies of The Journal-Record containing such article.

### THE MAYBRICK CASE AGAIN.

Mrs. Maybrick, the Alabama woman who was convicted of the murder of her husband by administering arsenic and who has been serving a life sentence in an English jail seems now to be in a fair way to be liberated by the clemency of Edward VII based on new developments in the toxicology of arsenic. A French scientist Gaulier has succeeded in isolating arsenic from healthy gland tissue and concludes that if not a normal constituent of the body, it is at least a frequent one gaining entrance through the medium of plants, vegetables and certain kinds of animal food, notably fish. Being veryslowly eliminated it tends to accumulate in the tissues and may in time reach a quantity sufficient to kill the host. It is on this assumption that a new view is taken of the Maybrick case and it may lead to her regaining her freedom after twelve or fifteen years of incarceration. There has always been good ground for the belief that her conviction was based upon insufficient evidence but all efforts-and they have been frequent and well sustained-to secure a pardon for her have been fruitless. It is hoped that the new discovery in relation to arsenic may not prove a bane instead of a boon as in the present instance.

